# Epidemiological trends and mid-term to long-term outcomes of acetabular fractures in the elderly in China

**DOI:** 10.1007/s00264-023-06032-0

**Published:** 2023-11-29

**Authors:** Gao Feng, Cui Tingrun, Ge Yufeng, Liu Gang, Tan Zhelun, Chen Yimin, Peng Weidong, Tu Chao, Bei Mingjian, Zhu Shiwen, Yang Minghui, Wu Xinbao

**Affiliations:** 1 Department of Orthopaedics and Traumatology, Beijing Jishuitan Hospital, Capital Medical University, Beijing, 100035 China; 2 Peking University Fourth School of Clinical Medicine, Beijing, 100035 China; 3 National Center of Orthopaedics, Beijing, 100035 China; 4 Medical School of Chinese PLA, Beijing, 100853 China; 5 Department of Orthopaedics, First Medical Center of Chinese PLA General Hospital, Beijing, 100853 China

**Keywords:** Acetabular fracture, Elderly patients, Retrospective study, Epidemiology, Fracture pattern, Outcome

## Abstract

**Purpose:**

To explore the epidemiological trends in acetabular fracture and report the mid-term to long-term clinical outcomes of the elderly treated with operation.

**Methods:**

Retrospective study. Patients aged ≥ 14 years with operative treatment of the Acetabular fracture from Jan 2010 to Dec 2019 at a level-1 trauma centre were identified to analyze the epidemiological trends, and the difference in fracture patterns between young and elderly patients (≥ 60 years old) were compared. The elderly patients were followed up to evaluate their clinical outcomes and satisfaction degree (worst to best: 0 to10). The patients were divided into the 2010–2014 group and the 2015–2019 group according to the year of admission, and the clinical outcomes of the two groups were compared to verify the stability from mid-term to long-term after surgery for acetabular fracture.

**Results:**

A total of 1024 patients (mean age 43.35 years, range 14–86 years) with acetabular fractures received operative treatment in this decade. The mean age of the acetabular fracture patients increased from 41.1 years to 47.7 years, and the proportion of elderly patients increased from 5.7% to 24.0%, with some volatility. The ratio of male to female decreased year by year, and the proportion of female patients increased with age. And the anterior fracture patterns were more common in the elderly patients compared to the young patients (*P* < 0.001). 118 elderly patients (82 males, 36 females; mean age 66.91 years, range 60–86 years) were followed-up (mean 77.4 months, range 35–152 months). The overall mortality rate of the elderly patients was 7.69% (9/118). The Harris hip score of those alive patients was 90.41 ± 12.91 points (excellent and good rate 84.4%). 87 patients completed the SF-12 with a normal HRQoL (PCS 50.49 ± 8.88 points; MCS 55.66 ± 8.86 points). 90.8% of the patients achieved a satisfaction score of 9 or higher. And there was no significant difference in clinical outcomes between the 2010–2014 group and the 2015–2019 group (*P* > 0.05).

**Conclusions:**

In conclusion, acetabular fractures presented an obvious ageing trend in China, and the fracture patterns of the elderly patients differed from those in the young patients. Operative treatment for elderly acetabular fractures yielded satisfactory and persistent clinical outcomes from mid-term to long-term clinical.

**Supplementary Information:**

The online version contains supplementary material available at 10.1007/s00264-023-06032-0.

## Introduction

In recent years, population ageing is accelerating worldwide, and the incidence of osteoporotic fractures (fragility fractures) is also increasing. It is reported that the incidence rate of acetabular fractures in patients aged 60 or older has increased 2.4 times in the past 25 years in America [1], and the elderly become the fastest growing subgroup of patients with acetabular fractures in the developed countries [2]. However, few studies have examined the ageing trend of acetabular fractures in China and other developing countries, and the long-term clinical outcomes of elderly acetabular fracture patients are lacking. This study retrospectively analyzed the clinical data of acetabular fracture patients at our hospital from 2010 to 2019, to explore the epidemiological trends in acetabular fracture in China and report the mid-term to long-term postoperative clinical outcomes in the elderly, in order to provide evidence for clinicians.

## Materials and methods

This is a retrospective study, and this study was approved by the Ethics Committee of our institution. The study objects were consecutive patients with acetabular fractures admitted to the Department of Orthopedics and Traumatology of our hospital, a level-1 trauma center, during the ten years from January 2010 to December 2019. Patient data were collected from the medical records and radiological documents to identify the patients diagnosed with acetabular fractures. Patients who met the following criteria were enrolled: ① Age ≥ 14 years old at the time of injury; ② Diagnosed by X-ray or CT; ③ The time from injury to admission is less than 21 days; ④ Received internal fixation or hip replacement; ⑤ Patients with pathological fractures or those who did not meet the above criteria were excluded. The patient data were collected retrospectively to analyze the epidemiological trends in age, cause of injury, gender ratio, combined injury, surgical approach and fracture pattern. And the difference in fracture patterns between young and elderly patients (≥ 60 years old) were compared.

The elderly acetabular fracture patients were followed up to get the clinical outcome information including survival, reoperation, mobility, hip function, health related quality of life (HRQoL) and satisfaction degree in 2022. The elderly patients were divided into two groups based on the year of admission, the 2010–2014 group (with long-term outcomes) and the 2015–2019 group (with mid-term outcomes), and the clinical outcomes of the two groups were compared to verify the stability from mid-term to long-term after surgery for acetabular fracture. Mobility was divided into 3 level: independent, walking aid, and non-ambulant. To facilitate follow-up by telephone, the Harris Hip Score (Self-Report) [3] was used to evaluate the hip function. Compared with original Harris Hip Score (HHS), the Self-Report version excluded the question on public transportation (1 point), and hip range of motion (5 points) and deformity (4 points). The other items, including hip pain (44 points), walking aid (11 points), limp (11 points), walking distance (11 points), climbing stairs (4 points), wearing shoes and socks (4 points), and sitting (5 points) were retained. The possible score range was 0 to 90. For ease of presentation, this score range was rescaled to 0 to 100. The Self-Report version of HHS was testified to have good consistency with the original HHS [3]. A score of 90 or more was considered excellent, 80–89 as good, 70–79 as fair, and less than 70 as poor. And the 12-Item Short Form Health Survey (SF-12) was used to assess HRQoL. It can be summarized as two comprehensive variables: Physical Component Summary (PCS) and Mental Component Summary (MCS). The final score ranged from 0 to 100 (worst to best), where ≥ 50 points represented normal. Scores ranged from 0 to 100, with 0 as the worst, 100 as the best, and a score ≥ 50 as normal. And the satisfaction degree is scored based on patients' subjective judgment (worst to best: 0 to10).

Continuous variables were presented as mean ± standard deviation (SD) and the categorical variables were presented as frequency (%). Student’s t-test were adopted to test for continuous variables, while Chi-square test was adopted to test for categorical variables. Statistical analysis was performed using the IBM SPSS Statistical Package (version 25) (SPSS Inc., Chicago, IL, USA). All statistical significance was established at* P* < 0.05.

## Results

### Epidemiological trends

**Fig. 1:**
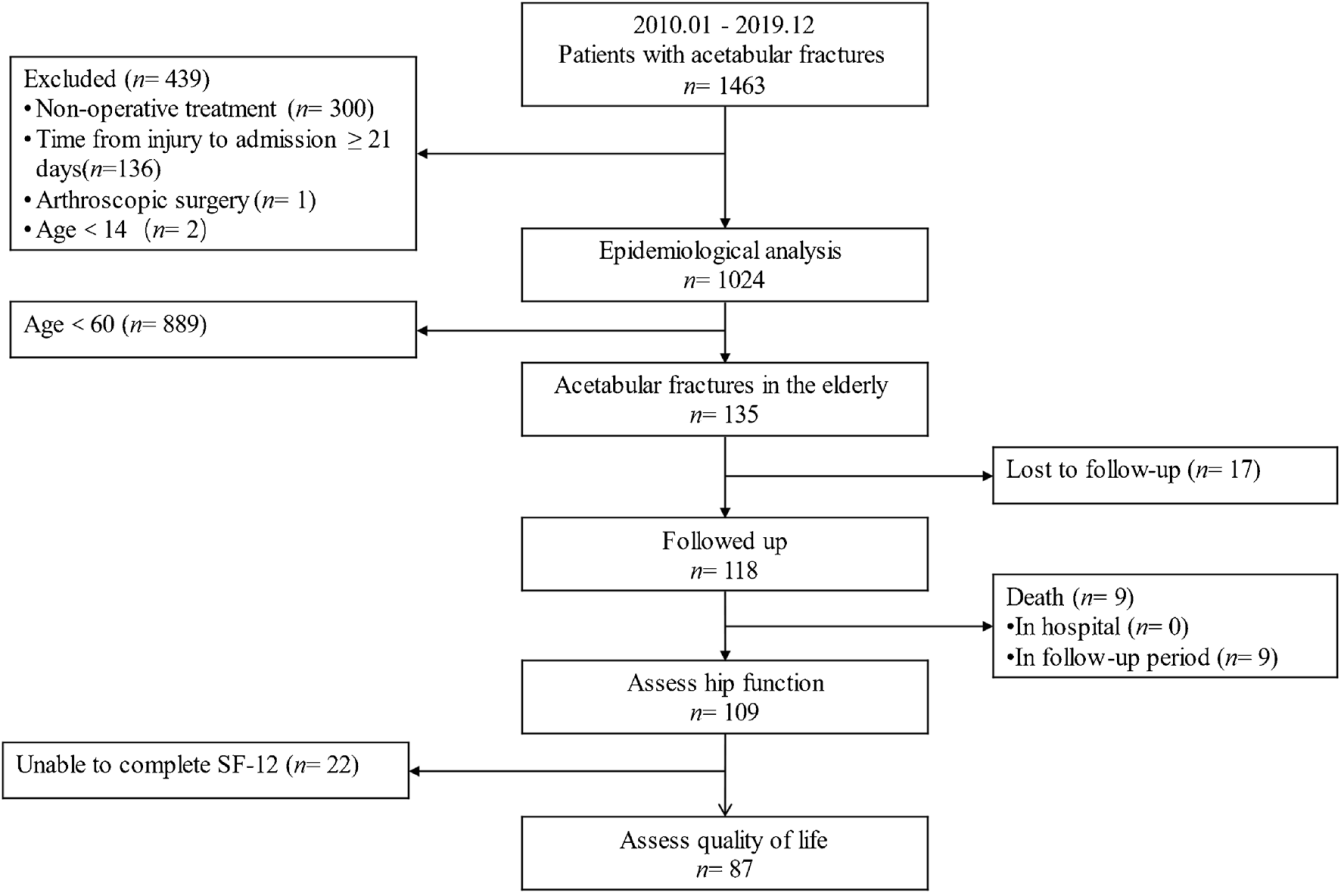
Flowchart of study

There were 1024 patients (mean age 43.35 ± 14.04 years, range 14–86 years) enrolled in this study to analyze the epidemiological trends in this decade, and 135 patients aged 60 or older, accounting for 13.2%. The flow chart of this study is shown in Fig. 1.

**Fig. 2:**
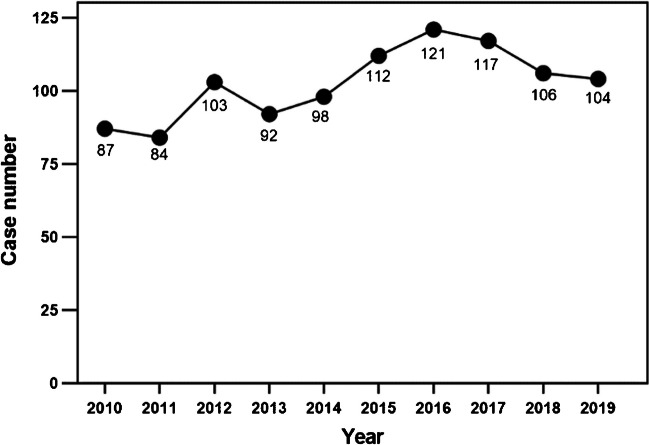
Trend chart of case number by years

**Fig. 3:**
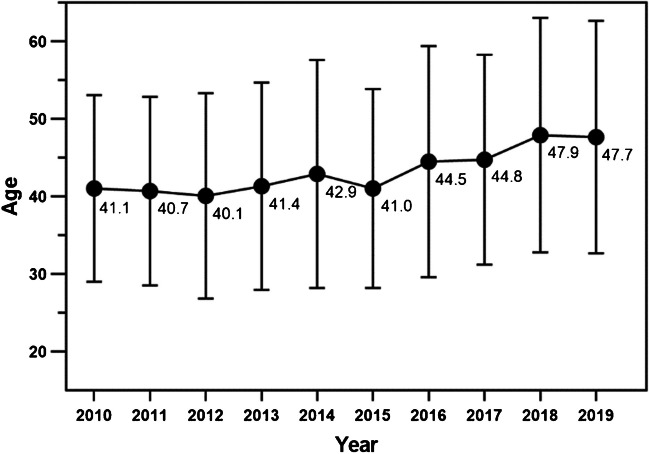
Trend chart of the mean age by years

**Fig. 4:**
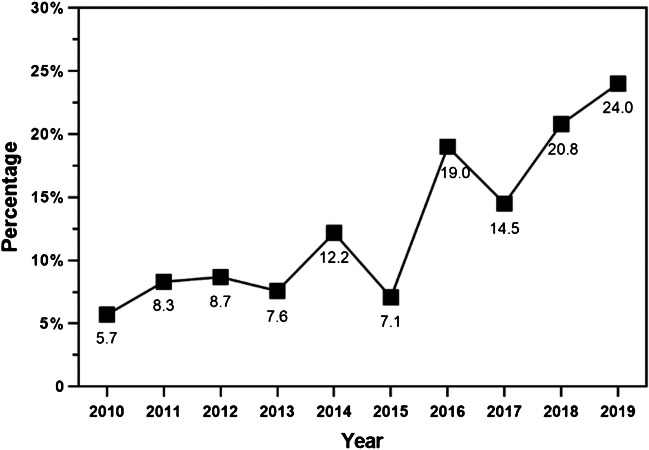
Trend chart of the proportion of elderly patients with acetabular fracture by years

The annual case number of acetabular fractures fluctuated slightly with the years (Fig. 2). And the annual mean age and changing trend are shown in Fig. 3, which significantly increased, with some volatility, from 41.05 ± 12.06 years in 2010 to 47.66 ± 15.01 years in 2019. The mean age of the second five years (45.10 ± 14.48) was higher than that of the first five years (41.23 ± 13.20), and the difference was statistically significant (*P* < 0.001). Furthermore, proportion of the elderly acetabular fracture patients changes over time (Fig. 4). The majority of acetabular fracture patients are young patients. And the proportion of elderly patients largely increased from 5.7% to 24.0% in this decade, also with some volatility. Proportion of the elderly acetabular fracture patients in the second five years (17.0%) was higher than that in the first five years (8.6%), with a statistically significant difference (*P* < 0.001).

**Fig. 5:**
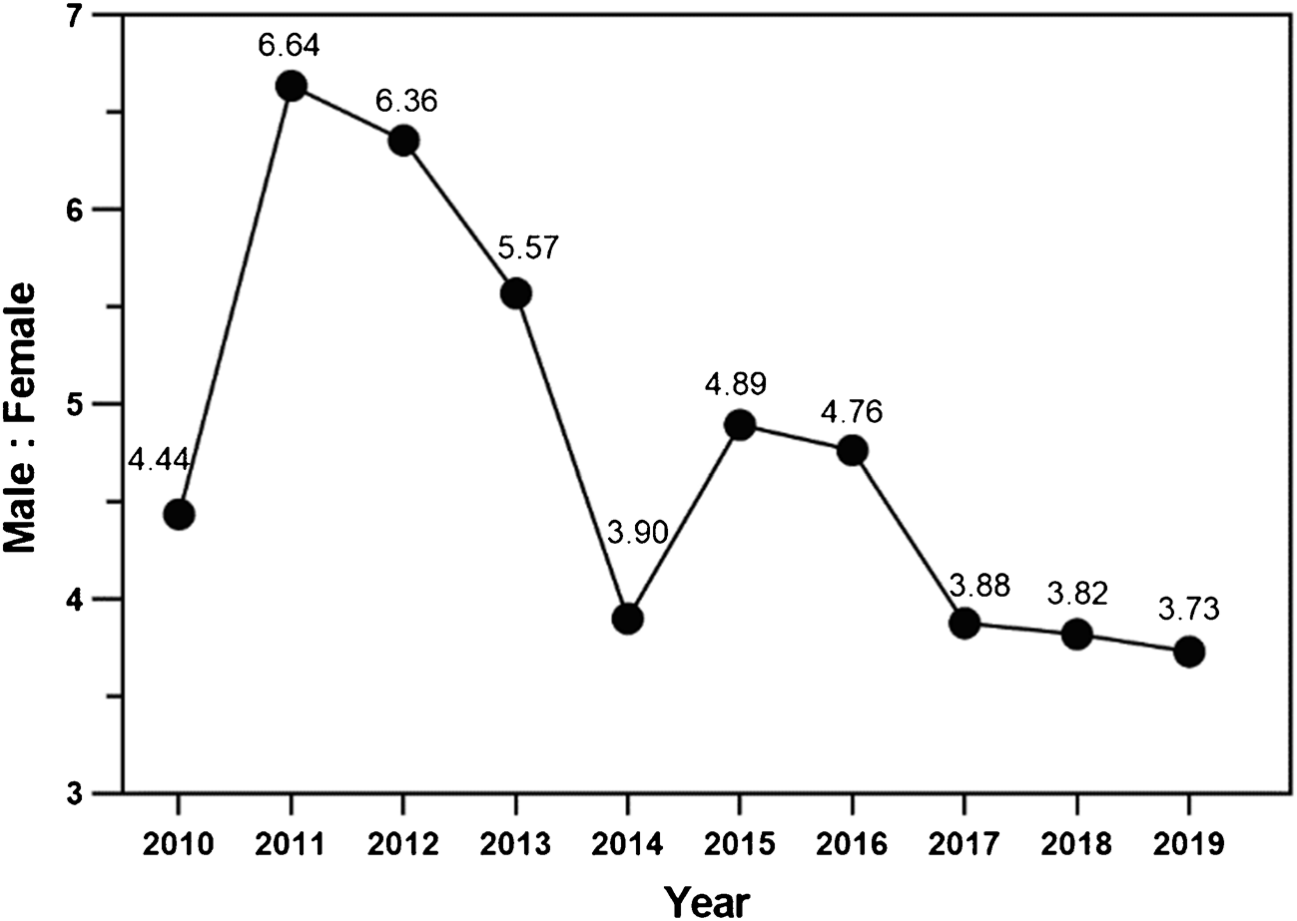
Trend chart of the gender ratio (Male: Female) by years

**Fig. 6:**
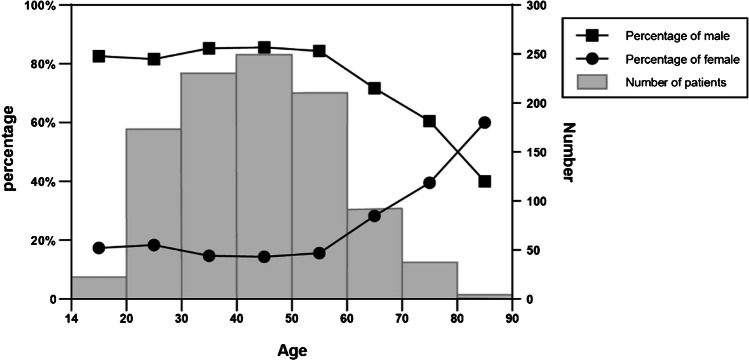
Trend chart of gender ratio by age

As to gender, the majority of acetabular fracture patients are male, but the ratio of male to female has changed over years (Fig. 5). Except for the two years of 2010 and 2014, the ratio of male to female showed a downward trend year by year, with the highest value of 6.64:1 in 2011 and the lowest value of 3.73:1 in 2019. But there was no statistically significant difference in the first five years and the second (*P* = 0.194). In addition, the proportion of female patients increases with age (Fig. 6). Of the patients aged under 60 years, 84.4% were male and 15.6% were female. In the elderly patients, 67.4% were male and 32.8% were female. The difference in the gender ratio between the two age groups is statistically significant (*P* < 0.001). And in the age group over 80 years, the proportion of female patients exceeded males, but there are only five octogenarians.

The difference between the 2010-2014 group and the 2015-2019 group in the cause of injury, combined injuries, surgical approaches, and fracture pattern was statistically insignificant (*P* > 0.05). The comparison of these factors in the first and second five years is detailed in Table 1.

**Table 1:** Comparison of patient and fracture characteristics between different admission time groups

	2010–2014 *n* = 464	2015–2019 *n* = 560	*P* value
Male, n(%)	389 (83.8%)	452 (80.7%)	0.194
Age of injury (years), mean ± SD	41.23 ± 13.20	45.10 ± 14.48	< 0.001
Cause of injury, n(%)			0.655
Traffic accident	279 (60.1%)	329 (58.8%)	
Fall	185 (39.9%)	231 (41.3%)	
Isolated injuries, n(%)	106 (22.8%)	146 (26.1%)	0.233
Combined injuries, n(%)			
Spine and pelvic	117 (25.2%)	129 (23.0%)	0.416
Upper limb	133 (28.7%)	173 (30.9%)	0.438
Lower limb	164 (35.3%)	207 (37.0%)	0.591
Head chest and abdomen	187 (40.3%)	217 (38.8%)	0.613
Approach, n(%)			0.897
K-L	275 (59.3%)	322 (57.5%)	
Ilioinguinal	110 (23.7%)	142 (25.4%)	
Stoppa	12 (2.6%)	14 (2.5%)	
Iliofemoral	0 (0%)	2 (0.4%)	
K-L + Ilioinguinal	52 (11.2%)	60 (10.7%)	
Other	15 (3.2%)	20 (3.6%)	
J-L classification, n(%)			0.465
Anterior column	22 (4.7%)	26 (4.6%)	
Anterior wall	4 (0.9%)	7 (1.3%)	
Posterior column	13 (2.8%)	18 (3.2%)	
Posterior wall	129 (27.8%)	172 (30.7%)	
Transverse	19 (4.1%)	22 (3.9%)	
Posterior column + posterior wall	5 (1.1%)	7 (1.3%)	
Transverse + posterior wall	114 (24.6%)	100 (17.9%)	
Anterior column + posterior hemitransverse	28 (6.0%)	36 (6.4%)	
T type	12 (2.6%)	23 (4.1%)	
Associated both-column	118 (25.4%)	149 (26.6%)	
J-L classification dichotomized, n (%)			0.659
Elementary	187 (40.3%)	245 (43.8%)	
Associated	277 (59.7%)	315 (56.25%)	
Total involving anterior displacement, n(%) ^*^	172 (37.1%)	218 (38.9%)	0.542

^*^ Anterior column, anterior wall, anterior column + posterior hemitransverse, associated both-column

*K-L* Kocher-Langenbach; *J-L* Judet-Letournel

Table 2 summarized the fracture pattern of acetabular fractures in the young patient and elderly patient group. The most common fracture patterns were associated both-column, transverse + posterior wall, posterior wall in the young group, and posterior wall, associated both-column, anterior column, anterior column + posterior hemitransverse in the elderly group. Compared with the young group, the anterior column, anterior column + posterior hemitransverse, posterior wall in the elderly group were significantly increased, and the transverse + posterior wall fractures were significantly reduced (*P* < 0.05). There were more elementary fractures and the anterior fracture patterns in the elderly (*P* < 0.001).

**Table 2:** Comparison of fracture characteristics between different age groups

	< 60 years *n* = 889	≥ 60 years *n* = 135	*P* value
J-L classification			
Anterior column	26 (2.9%)	22 (16.3%)	< 0.001
Anterior wall	9 (1.0%)	2 (1.5%)	0.964
Posterior column	26 (2.9%)	5 (3.7%)	0.824
Posterior wall	164 (18.4%)	37 (27.4%)	0.015
Transverse	37 (4.2%)	4 (3.0%)	0.508
Posterior column + posterior wall	9 (1.0%)	3 (2.2%)	0.431
Transverse + posterior wall	211 (23.7%)	3 (2.2%)	< 0.001
Anterior column + posterior hemitransverse	44 (4.9%)	20 (14.8%)	< 0.001
T type	27 (3.0%)	8 (5.9%)	0.881
Associated both-column	236 (26.5%)	31 (23.0%)	0.377
J-L classification dichotomized, n (%)			< 0.001
Elementary	262 (33.2%)	70 (51.9%)	
Associated	527 (66.8%)	65 (48.1%)	
Total involving anterior fracture patterns, n(%) ^*^	315 (35.4%)	75 (55.6%)	< 0.001

^*^ Anterior column, anterior wall, anterior column + posterior hemitransverse, associated both-column

*J-L* Judet-Letournel

### Clinical outcomes of the elderly patients

A total of 118 patients (82 males, 36 females) completed follow-up, with a mean age of 66.91 years (60–86 years). The follow-up rate was 87.4% (118/135), and the mean follow-up time was 77.4 months (35–152 months). In terms of treatment, 98.4% of the patients were treated with ORIF. Patient information was shown in Appendix Table 1. Combined injuries occurred in 55.9% of the patients, and the detailed combined injuries are shown in Appendix Fig. 1.

Nine deaths occurred up to the last follow-up, and the overall mortality rate was 7.69%. The age of death ranged from 64 to 86 years, and all were male and treated with ORIF. No in-hospital deaths occurred, and the earliest death occurred within 2 weeks after discharge. The Harris hip score of 109 alive elderly patients was 90.41 ± 12.91 points, and the excellent and good rate was 84.4%. At the last follow-up, 80.7% of the patients were able to walk independently, and 83.5% had regained their pre-injury mobility. For hip pain, 90.8% of the patients had no or only occasional hip pain without taking analgesics. 87 patients completed the SF-12, the mean PCS score was 50.49 ± 8.88 points and the mean MCS score was 55.66 ± 8.86 points. There were two cases of post-operative surgical site complications, including one case of incisional wound infection and one case of surgical site hernia. None of the patients underwent orthopaedic re-operation due to internal fixation failure or conversion to total hip replacement. And 90.8% of the patients achieved a satisfaction score of 9 or higher. And the difference in clinical outcomes between the 2010–2014 group and the 2015–2019 group was not statistically significant (*P*  > 0.05). Detailed clinical outcomes information and comparison of patients is shown in Table 3.

**Table 3:** Clinical outcomes of elderly patients

	Total	2010–2014	2015–2019	*P* value
Mortality, n(%) ^*^	9 (7.6%)	5 (14.7%)	4 (4.8%)	0.065
Age of death^**□**^	75.67 ± 6.73	74.0 ± 8.46	77.7 ± 3.86	0.443
Re-operation, n(%) ^*^	0 (0%)	0 (0%)	0 (0%)	-
Harris score, mean ± SD ^#^	90.41 ± 12.91	90.75 ± 12.98	90.28 ± 12.97	0.868
Harris score grade, n(%) ^#^				0.718
Excellent	73 (67.0%)	18 (62.1%)	55 (68.8%)	
Good	19 (17.4%)	7 (24.1%)	12 (15.0%)	
Fair	6 (5.5%)	1 (3.4%)	5 (6.3%)	
Poor	11 (10.1%)	3 (10.3%)	8 (10.0%)	
Mobility, n(%) ^#^				1.000
Independent	88 (80.7%)	23 (79.3%)	65 (81.3%)	
Walking aid	18 (16.5%)	5 (17.2%)	13 (16.3%)	
Non-ambulant	3 (2.8%)	1 (3.4%)	2 (2.5%)	
Regain pre-injury mobility, n(%) ^#^	91 (83.5%)	23 (79.3%)	68 (86.1%)	0.577
Pain of hip, n(%) ^#^				0.904
Slight or none	99 (90.8%)	27 (93.1%)	72 (90.0%)	
Severe	10 (9.2%)	2 (6.9%)	8 (10.0%)	
SF-12, mean ± SD ^∆^				0.105
PCS	50.49 ± 8.88	48.13 ± 10.65	51.51 ± 7.89	
MCS	55.66 ± 8.86	53.96 ± 9.80	56.38 ± 8.41	
Satisfactory, n(%) ^#^				0.320
10	88 (80.7%)	21 (72.4%)	67 (83.8%)	
9	11 (10.1%)	3 (10.3%)	8(10.0%)	
8	4 (3.7%)	2 (6.9%)	2 (6.9%)	
7	2 (1.8%)	1 (3.4%)	1 (1.3%)	
6	4 (3.7%)	2 (6.9%)	2 (2.5%)	

^*^ Only patients completed follow-up were included, *n* = 118

^□^Only death cases, *n* = 9

^#^ Only survivors to last follow-up were included, *n* = 109

^∆^ 22 patients unable to complete, *n* = 87

*PCS* Physical Component Summary; *MCS* Mental Component Summary

## Discussion

With the background of an accelerated ageing population, all types of fragility fractures are increasing [4], as well as the acetabular fracture [1, 5, 6]. It was reported that the annual mean age of acetabular fracture patients and the proportion of elderly patients in California from 1980 to 2007 presented an increasing trend, and compared with in the earlier period (1980–1993), the proportion of elderly people in the later period (1994–2007) of the study increased 1.4 times from 10 to 24% [1]. And the mean age of acetabular fracture was 66 ± 22 years in France (2006–2016) [6]. As for developing countries, it was reported that the mean age of acetabular fracture patients in India increased from 33 years in 2013 to 40 in 2019 [7]. And the mean age of patients suffering from an acetabular fracture between 2008 and 2010 was 36 years in Qatar [8]. In this study, the mean age in China was between the developed and other developing countries, which was parallel to China’s ageing degree. And the changing trends of mean age of the acetabular fracture patients and the proportion of the elderly acetabular fractures in this study is in line with the literature. Compared with the first five years (2010–2014), the mean age of the second five years (2015–2019) increased by approximately four years, and the proportion of the elderly patients almost doubled. The gender distribution of the elderly acetabular fractures patients is generally the same as that of patients in the entire age group, mostly male, and the proportion of female patients increased by years and age, which is correlates with previous studies [7]. However, there were more female patients than males among octogenarians. The reason may be that females live longer than males, and there were more elderly females than males in China.

For the elderly, a considerable proportion of acetabular fractures were osteoporotic (fragile) fractures caused by low-energy injuries [1]. When the patient with osteoporosis fell laterally, the force acted on the greater trochanter, and the forward and medial force was transmitted to the acetabulum through the femoral neck and head, which would break and displace the anterior column and quadrilateral plate of the acetabulum [1, 9]. Therefore, the anterior column-posterior hemitransverse fracture pattern was recognized as one of the classic osteoporotic acetabular fracture patterns [10]. In related studies [1, 11], both-column fracture was the most common acetabular fracture pattern in the elderly (23% ~ 26%); fractures involving the anterior column were the second most common, including anterior column-posterior hemitransverse pattern (15% ~ 19%) and anterior column fractures (11% ~ 19%), which were comparable to those in this study.

As for posterior wall fracture, it is the most common acetabular fractures pattern in adults (approximately 23%) [12, 13]. The main injury mechanism is that drivers with the hip and knee flexed are subjected to backward violence transmitted to the acetabulum from the dashboard in traffic accident [14]. In this study, the proportion of posterior wall fractures in elderly patients (27.4%) was significantly higher than the reported 8% ~ 13% in the literature [1, 11], it was also significantly higher than that of the younger group (18.4%), which may be attributed to the bias of the small sample size of the elderly patients in this study.

ORIF was the main surgical method for displaced acetabular fractures in the elderly in this study, and most patients obtained satisfactory clinical outcomes.

A systematic review [11] including 15 studies showed that the mortality rate of 203 acetabular fractures patients > 55 years old (mean age 69.5 years) after ORIF was 15.3% at a mean follow-up of four years. The mortality rate in this study was 7.6%, lower than in the literature. The difference may be due to the lower mean age in this study.

It was reported by Laflamme et al. [15] that the Harris hip score of the elderly acetabular fracture patients after ORIF was 87.9 points, with an excellent and good rate of 70.6%. And the Harris hip score after acute total hip arthroplasty (THA) was 70.4 points with an excellent and good rate of 59% [16]. The treatment of ORIF combined THA for the elderly acetabular fracture patients provided a Harris hip score of 88 points [17]. The Harris hip score in this study is better and the excellent and good rate is higher, which might be a result of that the patients in this study were relatively younger.

Only few studies using SF-12 to assess the quality of life in the elderly acetabular fracture patients after operation. The PCS score of the SF-12 for the elderly acetabular fracture patients after ORIF was 45.3 points, and the MCS score was 55.9 points in literature [15], which is roughly in line with this study.

For reoperation, Daurka et al. [11] reported that about 22.4% of the elderly acetabular fracture patients underwent secondary THA two to three years after ORIF. Many studies reported a high conversion rate of THA after ORIF for acetabular fractures in the elderly [11, 16, 18–21]. Therefore, acute THA is recommended if conditions permit. In this study, 98.4% of the patients were treated with ORIF. Although some patients had limited mobility and hip pain, no patient underwent acetabulum re-operation, significantly less than in previous studies [11, 16, 18–20, 22]. And this might be a result of the cultural difference and the lower functional requirements of the chinese elderly. The high satisfaction scores indicate that most patients achieve satisfactory mid-term to long-term outcomes after ORIF. In addition, the difference of clinical outcomes between the 2010–2014 group and the 2015–2019 group was not statistically significant, indicating that appropriate treatment for elderly patients with acetabular fractures can lead to relatively persistent hip function and quality of life from the mid-term to long-term.

This is the first study to reveal the epidemiological trends in acetabular fracture in China, and also report the mid-term to long-term postoperative clinical outcomes of the elderly. It will provide important evidence for the understanding and treatment of acetabular fractures in China and other developing countries. Also, this study possessed some limitations: ① It was a retrospective study, the information we collected was limited. ② The patients were only followed up by telephone, the physical and radiological examination could not be performed. ③ Hip function was assessed by the HHS (self-report). Thus, the outcome could not be compared with previous studies. ④ This was a single center study, the results of this study must be validated in larger multicenter studies with longer follow-up.

In conclusion, acetabular fractures suffered from a significant ageing trend in China, and the fracture patterns of the elderly patients differed from those in the young patients. Operative treatment for elderly acetabular fractures yielded satisfactory and persistent clinical outcomes from mid-term to long-term.

### Supplementary Information

Below is the link to the electronic supplementary material.

Supplementary file1 (DOCX 91.0 KB)

## Data Availability

The datasets used in this study are not publicly available because of patient confidentiality but are available from the corresponding author on reasonable request.
