# Supplementary material for: Epidemiological trends and mid-term to long-term outcomes of acetabular fractures in the elderly in China
Source: Int Orthop. 2023 Nov 29;48(2):563–72. doi: 10.1007/s00264-023-06032-0 (PMC10799810; doi:10.1007/s00264-023-06032-0)
Supplement: Supplementary file 1 — Supplementary file1 (DOCX 91.0 KB) [file 264_2023_6032_MOESM1_ESM.docx]

**Appendix Table 1** Information of elderly patients who completed follow-up

|  | Total  *n* = 118 |
| --- | --- |
| Male, n(%) | 82 (69.5%) |
| Age of injury (years), mean ± SD | 66.91 ± 6.02 |
| ASA grade, n(%) |  |
| 1-2 | 85 (72.0%) |
| 3-4 | 33 (28.0%) |
| Cause of injury, n(%) |  |
| Traffic accident | 64 (54.2%) |
| Fall | 54 (45.6%) |
| Dislocation, n(%) |  |
| Posterior | 29 (24.6%) |
| Central | 14 (11.9%) |
| None | 75 (63.6%) |
| Associated injuries, n(%) | 66 (55.9%) |
| Treatment, n(%) |  |
| ORIF | 116 (98.4%) |
| THA | 1 (0.8%) |
| ORIF+THA | 1 (0.8%) |
| Approach, n(%) |  |
| Koch-Langenbach | 48 (40.7%) |
| Ilioinguinal | 41 (34.7%) |
| Stoppa | 6 (5.1%) |
| Iliofemoral | 0 (0%) |
| Other | 23 (19.5%) |
| J-L classification, n(%) |  |
| Anterior column | 19 (16.1%) |
| Anterior wall | 0 (0%) |
| Posterior column | 4 (3.4%) |
| Posterior wall | 34 (28.8%) |
| Transverse | 3 (2.5%) |
| Posterior column+ posterior wall | 3 (2.5%) |
| Transverse + posterior wall | 3 (2.5%) |
| Anterior column + posterior hemitransverse | 16 (13.6%) |
| T type | 7 (5.9%) |
| Associated both-column | 29 (26.4%) |
| J-L classification dichotomized, n (%) |  |
| Elementary | 60 (50.8%) |
| Associated | 58 (49.2%) |
| Total involving anterior displacement, n(%) ^*^ | 64 (54.2%) |

ORIF, open reduction and internal fixation; THA, total hip arthroplasty; J-L, Judet-Letournel

^*^ Anterior column, anterior wall, anterior column + posterior hemitransverse, associated both-column.

**Appendix Fig. 1** upset plot of combined injuries
